# “This illness diminishes me. What it does is like theft”: A qualitative meta‐synthesis of people's experiences of living with asthma

**DOI:** 10.1111/hex.12605

**Published:** 2017-08-02

**Authors:** Kristen Pickles, Daniela Eassey, Helen K. Reddel, Louise Locock, Susan Kirkpatrick, Lorraine Smith

**Affiliations:** ^1^ Centre for Values, Ethics & the Law in Medicine University of Sydney Sydney NSW Australia; ^2^ Faculty of Pharmacy University of Sydney Sydney NSW Australia; ^3^ Woolcock Institute of Medical Research University of Sydney Sydney NSW Australia; ^4^ Nuffield Department of Primary Care Health Science University of Oxford Oxford UK

**Keywords:** adults, asthma, chronic illness, lived experience, qualitative synthesis

## Abstract

**Background:**

What matters to people in their everyday experiences of living with asthma is influenced by a diverse range of personal, social, medical and environmental factors. Previous reviews of the asthma literature have largely focused on medical aspects of asthma or specific population groups with particular needs.

**Objective:**

To identify, describe and synthesize from the available qualitative literature the views and experiences of adults living with asthma.

**Method:**

We systematically searched for qualitative studies reporting on the personal experience of living with asthma. A meta‐synthesis approach was used to analyse and interpret the data. Key themes relating to personal perspectives on asthma were identified and grouped into overarching concepts.

**Results:**

We identified 26 studies. There was a paucity of literature on the physical burden of asthma symptoms and the role of social support. Our synthesis generated a central concept of the “work” associated with living with asthma: work was of a personal nature, and at times an intensely emotional experience. Individuals tailored their behaviour in response to demands of the physical and social environment, including interactions with health‐care professionals.

**Conclusion:**

This is the first systematic review of the qualitative literature reporting on people's own perspectives of living with asthma. Our findings draw attention to the nuances and sensitivities surrounding patient experiences of self‐management. Medical care is a central plank of managing chronic conditions, but our health‐care systems are now expected to deliver patient‐centred care. Considering the broader aspects of asthma management, beyond that of symptoms and treatment, will help to facilitate comprehensive care.

## 1 INTRODUCTION

Asthma is a common chronic lung disease that cannot be cured. It affects as many as 334 million people of all ages in all parts of the world, causing an estimated 346 000 deaths annually.^1^ It is well established in the existing literature that asthma has a significant impact on physical, psychological and social well‐being, albeit with considerable variation between individuals. In 2010, asthma was ranked 14th in the world in terms of extent and duration of disability.^2^, ^3^ Asthma often interferes with daily living,^4^ is associated with poorer self‐assessed health status and is a substantial burden in terms of time off work.^5^ Much of this impact comes from the physical effects of asthma symptoms, but there is also a significant social and emotional impact. Previous studies have reported a high rate of mental health problems among people with asthma,^6^, ^7^, ^8^, ^9^ including anxiety and panic attacks affecting between 6.5% and 24% of people,^10^ a prevalence 3‐10 times higher than in the general population.^11^, ^12^ Further, the direct and indirect economic burden associated with asthma is one of the highest among chronic disease due to the significant health‐care utilization associated with this condition.^13^

Not surprisingly, clinical practice guidelines for the management of asthma mainly focus on medical aspects of treatment. Guidelines provide evidence‐based recommendations about diagnosis, assessment and appropriate use of medications and non‐pharmacological strategies, to minimize asthma symptoms and the risk of adverse outcomes such as flare‐ups and asthma‐related death. However, despite advances in medical care, poor outcomes for patients persist. Patient adoption of asthma self‐management practices, as recommended by health‐care professionals (HCPs), remains low. One factor may be discordance between patient goals for managing their asthma and those of the medical profession: an analysis of patients' personal goals found that 35% of those goals did not map to the medical elements of the 2006 Australian asthma guidelines.^14^

More recently, asthma guidelines have increasingly emphasized the need for patient‐centred care, taking into account the patient's perspective, finding out the patient's own goals for their asthma, and using shared decision making to engage the patient in a partnership to manage their asthma. This has shown to improve asthma outcomes and patient engagement.^15^ However, the extent to which a patient‐centred approach has been implemented in clinical practice is unclear. It is an on‐going challenge for both patients and health‐care professionals to discuss self‐management strategies in everyday consultations.^16^

One explanation could be a lack of research evidence about the patient's experience of living with asthma. The viewpoints of people about their day‐to‐day personal experiences of living with asthma, including its management, are extremely important. Reviews conducted so far have focussed on specific medical questions or population groups (eg, adolescents) rather than the broader personal experiences of adults living with asthma.^17^, ^18^ Currently, there is considerable disparity between research exploring the health priorities of people living with a range of chronic conditions, including asthma, and the management and treatment priorities of HCPs.^19^, ^20^, ^21^, ^22^, ^23^, ^24^, ^25^ This body of research reveals that HCPs focus strongly on asthma symptoms, their triggers and taking medicines, whereas people with chronic conditions are concerned with more personally relevant and broader lifestyle issues such as exercise, fatigue, sleep and stress reduction, with a lesser focus on disease‐specific problems such as use of medicines.^18^, ^26^ Personal and social factors, life goals and choice all appear to play a role in the ways in which people with chronic illnesses manage their condition. Exploring the perspectives of those most closely concerned—the people living with asthma—is an important step in furthering our understanding of the specific needs of those affected.

Published systematic reviews have examined specific aspects of asthma management, such as asthma action plans.^27^ However, there is currently no comprehensive systematic review (qualitative or quantitative) of the *personal experiences* of adult patients living with asthma. That is, what matters to people with asthma and how it has affected their lives.

Integrating qualitative research studies into a synthesis will generate new insights and understandings of the existent empirical work in this important area. The objective of this systematic review is to identify, describe and synthesize from the available qualitative literature the views and experiences of adults living with asthma. We ask the question, “How do people living with asthma experience their condition?”

## 2 METHODS

**Figure 1:**
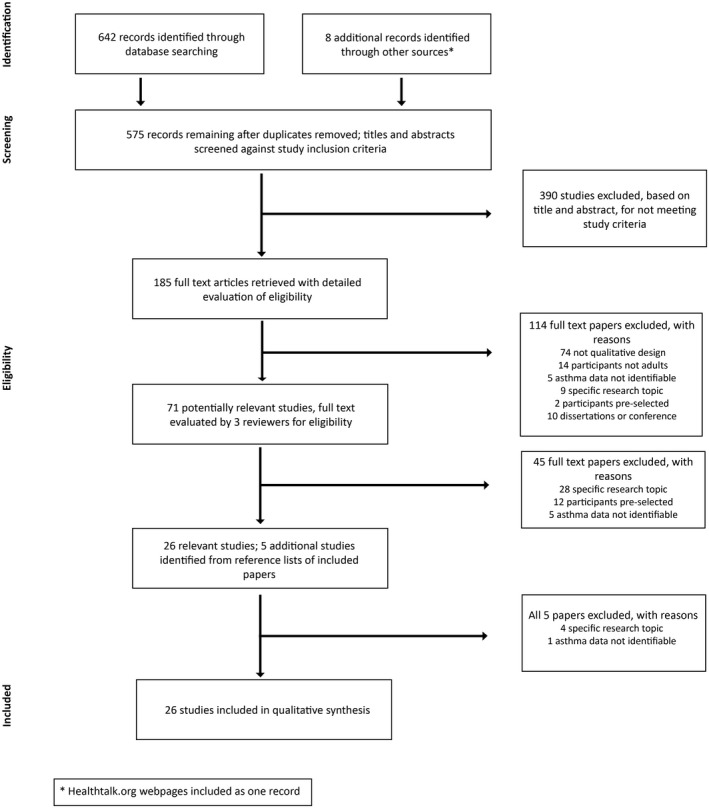
Flow of information through the different phases of a systematic review (based on PRISMA reporting flow chart)

We conducted a systematic review and synthesis of the qualitative evidence describing adult experiences of asthma. The review was reported using the Preferred Reporting Items for Systematic Reviews and Meta‐Analyses (PRISMA) statement (Figure 1).

### 2.1 Search strategy

A comprehensive literature search was conducted using the electronic databases: MEDLINE via OvidSP, PsycINFO via OvidSP, PubMed, CINAHL, EMBASE, Sociological Abstracts and Google Scholar for empirical studies reporting on the lived experience of adults with asthma (search strategy available in Appendix A), from inception to December 2015. Databases were selected due to their coverage of medical and allied health information. The journals *Qualitative Health Research* and *Qualitative Research* were searched using keywords relating to asthma. Additional references were sought by searching the reference lists of relevant studies. The review includes a study of experiences of asthma by the Health Experiences Research group, disseminated on the healthtalk.org website;^28^ each of its 25 topic‐based webpages were referenced separately.^29^, ^30^, ^31^, ^32^, ^33^, ^34^, ^35^, ^36^, ^37^, ^38^, ^39^, ^40^, ^41^, ^42^, ^43^, ^44^, ^45^, ^46^, ^47^, ^48^, ^49^, ^50^, ^51^, ^52^, ^53^ There is evidence for the appropriateness of including the healthtalk.org website as a reliable source of qualitative data. The website was previously used in a systematic review by the Evidence for Policy and Practice Information and Co‐ordinating Centre (EPPI‐Centre) on young people and obesity.^54^ The EPPI‐Centre review found the healthtalk.org website to be a highly rigorous and relevant source. In addition, it was the only study to cover all themes found in their review.^54^

### 2.2 Study eligibility

Studies were included if: (i) the article reported on the personal experience of adults living with asthma. We focused on the literature on adult experiences only because the experiences of children with asthma would be reported mainly through their parents, and adolescents' experiences are distinctly different from those of adults or children;^55^ (ii) the data collection and analysis methods were reported as qualitative; (iii) the publication reported original primary data; and (iv) the publication was in English language only, due to lack of resources for translation. This review considered studies that focused on qualitative data or included a qualitative aspect, including, but not limited to, designs such as phenomenology, grounded theory, ethnography, illness narrative and action research. We excluded (i) studies that focused on a specific medical question rather than the personal experience of living with asthma, (ii) studies where participants were preselected by their inclusion in another study, because of the potential for sampling bias or modification of their experience, and (iii) studies that were specifically about the experiences of living with “severe asthma”; this condition, affecting around 5%‐10% of the asthma population, is characterized by failure to respond to optimized conventional asthma treatment, and the diagnosis is established only following specific detailed investigations.^26^ For the same reason, data from interviews in healthtalk.org^28^ with patients who were clearly identifiable as having severe refractory asthma were also not included.

We identified 575 reports of which 26 met the inclusion criteria for the synthesis (Figure 1). Results were merged using reference management software (Endnote), and duplicates were removed.

### 2.3 Data extraction and analysis

Two reviewers (KP and HR) extracted the data onto a data extraction form for qualitative analysis. The data extracted from the studies included specific details about study location, date of publication, qualitative method, participants, recruitment (primary or secondary care), study aim(s), methods for data analysis, principal experience(s) explored, diagnosis and definition of asthma, medications and duration of diagnosis.

This study was a meta‐synthesis of the qualitative literature. We used this systematic and iterative method to integrate themes and synthesize results across the studies into taxonomies detailing the range of conceptual findings. The synthesis was led by KP and LS. Views reported by participants in the original studies, including the authors' interpretations of those experiences, were extracted verbatim and became the data for the synthesis. We compiled tables summarizing the main themes, and conclusions drawn, about people's experiences of asthma, as reported in the papers. Themes and concepts identified in the original studies were examined in relation to one another and across the studies. The reviewers went back and forth between the original papers, the extracted data and the tables of reported concepts and themes continually during the analysis. We constructed codes and categories iteratively from the text to capture the meaning and content of the participants' perspectives. Preliminary interpretations were presented to the wider team for critical analysis and refinement. By considering the data within the framework of our research question—”How do people living with asthma experience their condition?”—we explored the possibility of more abstract or analytical themes or concepts. The process culminated in the development of a “line of argument,”^56^ bringing the range of conceptual findings together and, crucially, going beyond the content of the original studies.

### 2.4 Quality assessment

Criteria for assessing the quality of published qualitative research are contested.^56^ Some authors question the appropriateness of using structured quality appraisal tools to assess qualitative research because of the diversity of approaches in collecting, analysing and interpreting qualitative data.^56^, ^57^ There is little empirical evidence in relation to excluding qualitative studies based on quality assessment, and quality appraisal guidelines and checklists do not necessarily produce greater consistency of judgements about which studies to include in a qualitative synthesis.^58^ Concerns have been expressed about less well conducted, but more insightful studies being excluded from qualitative syntheses.^58^ Accordingly, and like authors of previous qualitative syntheses,^59^, ^60^, ^61^ we considered the quality of the studies on the basis of their conceptual contribution, that is the relevance and usefulness of their findings to our research question.

## 3 RESULTS

From 575 potentially relevant studies, 26 satisfied our inclusion criteria (Appendix B). The included papers were published between 1993 and 2014, from the United States (8), Australia (7), United Kingdom (7) and Denmark, Portugal, Turkey, Canada and the Netherlands/Canada (one each).

Participants were mostly recruited from hospitals or hospital emergency departments (number of studies=11) followed by primary care (number of studies=5). The favoured qualitative method was semi‐structured interviews, and data analysis was mostly thematic.

The number of participants included in the studies ranged from 4 to 95 people. There was a considerably higher proportion of female participants, consistent with asthma being more prevalent among females than males in the adult population.^3^ Participant age varied from 18 to 73 years (not all studies reported patient age). Eight studies specified the inclusion of a minority group, predominantly African American.

The included articles reported on a range of contexts relevant to individual experiences of asthma: thirteen reported on asthma management,^40^, ^41^, ^45^, ^62^, ^63^, ^64^, ^65^, ^66^, ^67^, ^68^, ^69^, ^70^ six articles on patient experiences of emergency care,^42^, ^71^, ^72^, ^73^, ^74^, ^75^ eight articles about asthma medication use and treatment,^37^, ^38^, ^39^, ^44^, ^76^, ^77^, ^78^, ^79^ four articles about patient and physician communication,^43^, ^53^, ^80^, ^81^ two articles on quality of life,^82^, ^83^ one on asthma and the workplace,^47^ seven on general experiences of asthma,^46^, ^48^, ^50^, ^51^, ^84^, ^85^ two articles on asthma and lifestyle issues^49^, ^86^ and six on the symptoms and causes of asthma.^30^, ^31^, ^32^, ^33^, ^34^, ^35^

The included studies varied in design; both study design and content were considered to be of reasonable quality and of direct relevance to this review.

From the 26 studies, 64 experiences were extracted and grouped into 20 categories. Four synthesized concepts were produced from these categories:

1. Individual experiences of asthma are shaped by diverse physical and social environments (Table 1);
2. Individuals tailor their behaviour to their immediate context (Table 2);
3. Living with asthma necessitates on‐going periods of cognitive and emotional adjustment (Table 3); and
4. Living with asthma involves both positive and negative interactions with the health‐care system (Table 4).

**Table 1:** Synthesis 1: Individual experiences of asthma are shaped by diverse physical and social environments^a^

Categories	Findings—the experience
The physical burden of asthma	Physical symptoms can be frightening “panicky,” “choking,” “I was fighting”^65^, ^82^ “breathing through a straw,” “suffocating,” “drowning”^34^
	Asthma symptoms are unpredictable “…Because some people you can [imitates someone gasping with an asthma attack] but sometimes I don't get that, I just have a very, very, tight, tight chest. And that's all the symptom I can get. So I'm not always, for the medical profession they have a sort of a check list…but not everyone meets that check list criteria every time.”^34^
	Diminished capacity “I want to take a deep breath comfortably. I want to run. You suffocate me when you are inside me”^85^ “It was terrible. I, I could not walk across a normal living space…I couldn't really live a normal life. I mean, when my asthma was bad it was just so bad I really, I, actually I didn't want to live because I just couldn't do anything.”^32^
Feeling judged by others	Feeling judged by family member “It's all in your head, mum”^70^
	Feeling judged by society “I do worry about what other people think about my asthma…I become concerned that they'll think, ‘Oh my goodness we've got somebody with asthma, what are we going to do?' And actually I just want to say to them, I'm fine and I can manage this. And I'll let you know if I'm not.”^51^
	Feeling judged by employer “You must be on the bitch medicine again”^78^ “this guy's going to be sick all the time, so we won't bother”^47^
	Feeling judged by health professional “When I was at the hospital…the doctor said ‘You're not sick, go home…' I didn't go there because I had a little scratch on my finger”^67^
	Experience overruled by medical authority “I tell him it's [medication] not working and he tells me he's the doctor”^80^
Judging oneself	Feeling guilty when wanting space from children due to asthma
	Questioning legitimacy of experience “It is illegitimate to call in sick because of asthma. It's just silly, right”;^84^ “Am I wasting everybody's time”^63^
	Delay seeking help because asthma not a “serious” condition “I call the ambulance… when my lips are blue; You don't feel sick enough if you call one [ambulance] yourself”^64^
	Feeling embarrassed about having asthma “I find it embarrassing to even have asthma”;^70^ “you think you are sort of decrepit if you're asthmatic”^76^
Age of diagnosis	Childhood diagnosis “It was round when I was about ten, I was playing sport. I used to play a lot of sport when I was younger. I still do now…Initially when I was to go back to it, I found it hard to breathe…and just, just wasn't able to keep up really. And then …your body does adjust even with the inhaler, you get used to it and you find yourself being able to sort of compete with everyone else, being as fit as everyone else.”^31^
	Adulthood diagnosis “I was really shocked [about being diagnosed with asthma], ‘cause I just thought ‘how can someone as fit as me get asthma?' [laughs].”^32^ “I realised that it wasn't just a matter of fitness, it was actually a medical condition that I had then. And that was when I had to, kind of admit to myself that I was a wheezy person…And I was really anti‐medicine. So I found it quite hard to take on board that I was an ill person that needed to take medicine…”^32^ Childhood diagnosis “it's [asthma] become second nature…once you get into it…you find yourself being able to sort of compete with everyone else…”^31^ “There are going to be anger management issues there and resentment that I don't so much have because… I've not known life without it.”^32^
Learned responses	Conditioning: learning from family experiences “smacked” as a child “for keeping people awake” [with her chronic cough];^65^ “It was always [name] and her cough ‐ we all just got used to it”^69^
	Conditioning: learning from personal experiences “Asthma is part of me like my bad temper”^76^

a The categories and themes reported in the following tables represent a synthesis of those reported by the authors of the original studies.

**Table 2:** Synthesis 2: Individuals tailor their behaviour to their immediate context

Categories	Findings—the experience
Concern about possible judgement from others	Disassociating oneself with “asthmatic” identity
	Selectively disclosing asthma status “You have to be careful who you tell”^76^
	Choosing not to talk about asthma with others “I would not just talk about my asthma with others…I don't want them to think I am a pussy”^84^
	Not participating in activities likely to trigger symptoms in public or inconvenience others “I usually do not take a walk with others because I don't want to slow them down”^84^
	Concealing taking medication in social situations “If you are with strangers it is embarrassing to take medication in front of them…I do not take my medication in unknown territory”^84^ “Sure you have to take your inhalers from time to time, and sometimes, I guess when you go to a new place for instance you join a new club or you're with a new group of people there is a factor where you don't want them to see you blowing on the inhaler, because it looks quite geeky. It looks, it doesn't look too, too cool.”^37^ “I don't know why. It was just like, it's almost an admission of weakness [using a reliever], you know, there's something…. You know, I don't want to be seen as sickly as weak you know, but it's not, you know, you're not really sickly with it, it's just something that's just gone wrong.”^37^
	Concealing taking medication in the presence of employers
Normalizing condition and experience	“Soldiering on” “I come out of hospital and go straight back to work……if I'm fit enough to be out of there I'm fit enough to be at work”^64^
	Avoiding activities that will compromise employment
	Asthma is routine As “routine as putting one's watch on in the morning”^76^ “That's just so important, it's just part of the morning and night routine, before you clean your teeth, you take your inhaler, breathe in, wait for ten seconds breathe out, if you need another dose you take it.”^41^ “You can always see there's someone with the same symptoms as you or, you know, if not worse than you kind of thing. And that's that is one of the things I think about online forums is that they can be quite reassuring in the sense that you're not the only person that's living with this condition and these symptoms…”^45^
Taking proactive measures	Seeking information
	Planning or anticipatory actions “I used to go to bed with my shoes on, it used to be so bad. Now at least I put a pyjama top on”^71^
	Becoming computer literate to research asthma
	Obtaining a personal peak flow meter to monitor asthma^72^ “I measure my peak flow, that's the volume that you're able to inhale and exhale. I measure that usually three times a day and keep records of it…And if my peak flow shows that I'm sort of 20% below what I ought to be, or below my sort of personal best in terms of peak flow output then I start using prednisolone.”^41^

**Table 3:** Synthesis 3: Living with asthma necessitates on‐going periods of emotional and cognitive adjustment

Categories	Findings—the experience
Managing asthma by denying	Denying asthma diagnosis “I don't know if I could cope if I really had it”;^76^ “Well the doctors say I've got asthma but I've been hard to convince of that actually”^69^
	Minimizing experience/condition “I've got a bit of a cough but it doesn't mean I go to an asthma clinic. I wouldn't belong”;^76^ “I'm not sick enough”^84^
	Denying need to take medications Taking medication every day *“*it's really admitting to yourself that you are an asthmatic with a real problem here”^76^
Fearing the associated implications of having asthma	Fearing dependency on medications
	Fearing or experiencing side effects from medication “…I'm not exactly a slim person in the first place, and that's very hard to control, not wanting to eat more, and that's something that I find very hard. But, it's [taking oral steroids] the lesser of the two evils.”^38^ “I didn't like it at the start because it's almost like just, I mean I think in everybody's head there's a kind of a, don't go there factor with taking steroids because it feels like you're, you know, polluting your body in some way”^38^
	Fearing unpredictability of asthma “I feel worried about the places I visit, my health, and the possibility of an asthma attack all the time”^85^ “… Asthma is an absolutely awful condition because we all take breathing for granted until you can't breathe. To not be able to breathe and …it can also be painful for your chest is terrifying, and you just sometimes think, ‘Well, if the next one doesn't kill me the next one might…I'm not enjoying life right now'. And my mate died in the holidays ‘I really wish this would end' because you're also made aware that there's no cure.”^31^
Feeling disappointed about having asthma	Making sacrifices “This illness diminishes me. What it does is like theft.” “It means growing away from the pleasures of life”^85^
	Wishing to be “normal” “I wish I could be normal like everybody else; Why can't I be like others, just walking normally and nothing happening to me?”;^86^ “I hate feeling like I'm different”^68^
	Being significantly restricted and fighting those imposed restrictions, lacking achievements
Finding meaning	Asthma is “horrible,” “gloomy”^85^
	Comparing asthma experience to other chronic conditions “Asthma is not the worst thing to get”^84^
	Wondering, why me? “I used to ask myself ‘Why me?' …After learning to live with it, as I grew up, I tried adapting it to my life standards instead of seeing it as an obstacle to my moves”^85^
	Having asthma takes time to come to terms with “You can't in the beginning, especially before diagnosis, because you haven't, you might have no idea why you're ill. Why you feel like you have no energy, why you can't do certain things, why you can't do certain jobs. You career can be affected by it. Your home life is affected by it. Your social life is affected by it. And I think people who are newly diagnosed have got to give themselves time to come to terms with it”^46^
Acknowledging loss	Losing particular roles; loved sport or pets
	Cannot be in some public spaces “I can't go to a public place, someone might have perfume or smoke a cigarette and then I become endangered”^86^
	Choosing flexible employment
	Reluctantly adapting to restrictions and lifestyle change
Becoming expert on asthma—knowing what to do	Experiencing significant pivotal episodes “I never have carried medication before, and I probably always will from now on…now that I've come that close to having to go to the hospital”^71^
	Developing personal disease experience “I think I understand my asthma now, and it's like when I was ill on Saturday I knew, you know, how much I could take of that environment before I'd got to remove myself from it.”^41^
	Discovering limits to self‐control “I knew it was beyond my ordinary medication routine…it's time. You better get the hell out of the house, you know, get to the doctor.”^71^
Acceptance	Accepting symptoms and dependency on medication
	Accepting identity “You just have to find your way with it. And then try and get on with life and accept that you will have bad days…. I work full‐time. I'm off sick less than healthy people because I manage it. I travel abroad on my own. It's not going to stop you from having a life, you just have to… find the life that suits.”^46^
	Accepting inconvenience—“it's nuisance value” but a small price to pay
	Never been affected by asthma badly and do not look upon it as an illness “I am not bothered by my asthma; it's irrelevant”;^76^ “never really thought about asthma. I just had to live with it”^84^ “So I just hope that anybody who is watching this and is worried, it's worth just beating it. It's a long term, at the moment a long term and what they call a chronic condition which I hate, I hate the terminology, but it doesn't mean to say that it must rule your life.”^41^

**Table 4:** Synthesis 4: Living with asthma involves both positive and negative interactions with the health‐care system

Categories	Findings—the experience
Receiving different quality health care	HCP has poor knowledge about asthma and medications “My GP is a good doctor but he does not know much about asthma”^67^
	Preferring outpatient care [rather than GP]; outpatient staff know what to do
	Not given choice to participate in health care; no time available “They give you what they want to give you”;^80^ “All they do is give you tablets…take that and go away”^72^
	Experience dependent on HCP “He [His GP] he's actually an asthma sufferer himself… but he's also reassuring at the same time… he just explained how it hasn't hampered his life…He stays fit and healthy; it's again, positive mindset.”^43^
The importance of communication and understanding	Not being heard “Some of them [HCPs] don't want to listen”;^80^ “the doctor was just kind of oblivious…we never actually sat down and made a plan”^68^
	Receiving insufficient information and advice “My GP gave me a prescription for an inhaler but didn't tell me how to use it”^66^ “…he prescribed the preventer and the Salbutamol or Ventolin…And the next time I saw the GP, because I asked for an explanation and she said, ‘Oh because you have to'. And she didn't go into details and I'm the sort of person, I like to understand why I'm taking something. Not just because I have to. I need to understand the reason for it. So it helps me to appreciate the seriousness.”^43^
	Asthma management explained well in outpatient clinic
	Disagreeing about medical care “So me and the doctor, we couldn't agree about It”^67^
Tailoring adherence to recommended prevention and treatment	Being limited by inability to afford medications—tailor to suit or go without “I was short of money…so I went without Seretide [a long‐term inflammatory medication] for a week and that was a big mistake because I ended up going back in [to hospital]”^64^
	Asthma medication can be expensive—purchase but with compromises “I can't afford it [Flixotide, a long‐term anti‐inflammatory medication] financially…I'm trying to compensate by using more Ventolin, more Atrovent [both short‐term reliever medications], or something like that. So this has an effect on me too”^83^
	Being limited by inability to access health care
Preferring to manage asthma independently	Preferring to self‐manage “I understand my asthma better than anyone else. Someone that lives with it. So if they [doctor] don't answer me the way I know they should be I won't go back”^72^
Health care is a necessity	Not wanting to end up in hospital “…I was hospitalised and nebulised and treated with prednisolone for another attack and that was because I misjudged how far down into breathing difficulties I was because I didn't have a peak flow meter.”^44^
	Taking medications (reluctantly) to feel in control “I don't like taking steroids…but here. I need them to breathe properly, so what can you do?”^76^

We present the summarized results in Tables 1, 2, 3, 4. Each table represents collective observations we derived from looking across all studies; they are our interpretation of the evidence. The “categories” were extracted from the data, either in our words or according to how the primary studies categorized the data. The “findings” column presents the experience of asthma as described in the primary studies. Participant quotations extracted from the original studies illustrate the final four core concepts.

### 3.1 Synthesized line of argument: “work”

Our synthesis of the findings culminated in a line of argument about the active and passive “work” of living with asthma: “passive” being the more routine aspects of the experience itself, and “active” comprising efforts by the patient to present as living normally or actively defending against asthma. The “work” was at times an intensely emotional experience. A variety of cognitive and behavioural strategies were utilized by people living with asthma to negotiate the demands of their immediate environments, including interactions with health‐care professionals (HCPs).

#### 3.1.1 Individual experiences of asthma are shaped by diverse physical and social environments

As presented in Table 1, some participants judged themselves according to their experience of asthma, and questioned the legitimacy of that experience. Personal experiences were greatly shaped by everyday interactions with the external environment. Some environments were more physically, socially and emotionally challenging to “work” within than others. For example, some participants described delaying seeking health care because they had been conditioned to feel embarrassed by their asthma, while for others asthma was just another part of their personal make‐up.

Of note were the participants who reported experiencing negative interactions, judgement or de‐valuing of their personal experience by employers, family and health‐care professionals; “when I was at the hospital on the Friday, and the doctor said ‘You're not sick, go home'. … Yes [I felt judged]. Very much, and very much lately. Oh yes. We've seen it more and more over the last couple of years. I often wait, I wait until the last minute*”*.^67^ For some, those situations served as concrete cues prompting them to question how they relate to their asthma, and subsequent efforts to work with or against their asthma.

#### 3.1.2 Individuals tailor their behaviour to their immediate context

For some people, living with asthma was an on‐going process of monitoring and appraising themselves, others and their environment for relevant cues, and tailoring their behaviours responsively—according to the messages they received from that immediate context and as we observed, in notably self‐protective ways (Table 2).

Many of the findings of this review indicated that people felt it important to conceal their condition and/or medication use as a means of self‐protection from undesirable consequences, such as negative social judgement, “there's a certain amount of the public have a certain amount of baggage about asthma*”*.^83^

#### 3.1.3 Living with asthma necessitates on‐going periods of emotional and cognitive adjustment

Our synthesis draws attention to the idea that living with asthma is an on‐going “work in progress.” The concept of work was not necessarily readily observable, rather “work” of a personal nature, including emotional adjustment and shifting cognitions (Table 3). For example, finding meaning and acknowledgement of loss represented substantial adjustment to changed and changing circumstances; “I'd love to be able to walk … go on a holiday. If I go anywhere, I go out in the car, I've got to think … where's the hospital from there, where's the doctor from there … it's just been horrendous*”*.^83^

Our data suggest that living with asthma can be a variable emotional journey. Adjusting to asthma appeared to be a process that evolved via learning and responding to varied circumstances through lived experience. As the natural history of asthma is characterized by variability in its symptoms over time (symptoms may be present or absent at any given moment), individual trajectories varied too: coping and adjusting involved harder work at different points in time, including the emotional impact of the adjustment. Denial was a core experience illustrating the personal and often emotional work involved. This was exemplified through people's belief that they did not actually have asthma, or through efforts to minimize the effect of the condition, or a tension between the need to take medications every day and identification with being a person with asthma.

#### 3.1.4 Living with asthma involves both positive and negative interactions with the health‐care system

Dissatisfaction with primary care was a prominent experience, and an interesting finding given the emotional burden described. Unfortunately, for some individuals, their asthma experience was not validated by health‐care interactions; “some of them don't believe that you're sick;^80^ they just placate me … and act like, ‘what did you waste my time for'”.^71^ Some data suggested dismissive and negative judgemental comments from HCPs (Table 4). An inability to afford asthma medications and treatment was also a relatively common concern and in some cases influenced patient interactions with health‐care providers; *“*I got cut off my benefits and I couldn't afford medication…had an attack and no medication. They [HCPs] just thought it was my neglect, but I just didn't have any money to buy anything*”*.^73^

## 4 DISCUSSION

Asthma is a heterogeneous condition; the individual experience of it inevitably varies from one person to another, and within individuals too as symptoms and personal circumstances fluctuate. Our synthesis captures the multiplicity of the experience of asthma as a chronic condition and highlights the significant contribution of everyday interactions to that individual variation.

The majority of the evidence related in some way to the work of appraising one's personal situation, adjusting to, and for some patients, acceptance of their experience. This resonates with the wider literature on biographical disruption and repair in chronic illness, as people reflect on what has changed in their sense of self, what remains the same and what can be regained.^87^, ^88^, ^89^, ^90^, ^91^, ^92^ The episodic nature of asthma, particularly in its milder, well‐controlled form, may result in little sense of loss of self, and may indeed lead people to reject any on‐going illness identity, unlike those with enduring or progressive illness. By contrast, those with severe and perhaps very visible asthma may experience a more lasting sense of disruption and a continuing awareness of vulnerability to a lethal episode.

This review adds considerable depth to what has already been highlighted in quantitative studies. For example, we know that depression and anxiety are common among people living with asthma, but while standardized psychological measures provide important information about the health and well‐being of people with asthma, these quantitative measures often miss the subtle complexities of the actual patient experience. This synthesis highlights a broad range of emotional experiences, including key factors driving those experiences at the individual level. Personal experiences streamed from and were shaped by external influences, such as local contexts and interactions with health care.

Interpersonal relationships and social environments play an important role in shaping people's life‐experiences. Yet in this review, we noted very few studies reporting on the social support systems of people living with asthma; there were few data in relation to family or friend relationships or other social networks and their place in this context. Only one study appears to have directly asked people with asthma about the support they receive from friends, family and other support groups.^28^ In addition, this was also the only study to contribute to all themes synthesized in the review.^28^ Given the impact of asthma on daily routines and responsibilities for many people, we presume that emotional support could form an integral part of living with and managing asthma, and when present, could offer benefit to lessening the overall workload of living with asthma, particularly its emotional impact. The extant literature suggests a positive relationship between social support and asthma self‐management behaviours,^93^ and asthma control and quality of life.^94^, ^95^ However, in this review, our findings highlight that when social contacts or networks were reported, it was mostly in the context of exerting *negative* influence and creating social vulnerability.

The relative absence of descriptors of the physical experience of asthma in our review was also notable, except in the Healthtalk resource.^28^ Asthma is defined clinically as the combination of variable respiratory symptoms (eg, wheeze, shortness of breath, cough and chest tightness) and excessive variation in lung function. Its treatment is focused on minimizing symptoms, reducing the risk of flare‐ups and asthma‐related death and improving quality of life, including the use of various medication regimens, depending on disease severity. We identified only a small number of studies reporting on patient descriptions of the physical experience; these descriptions often encapsulated the fear and panic experienced by some people living with asthma. A 2006 study reported 53% of patients believed they had asthma only when they were experiencing physical symptoms.^96^ This begs the question, was the overarching emotional experience of most participants included in the present review a more prominent feature of their condition because physical symptoms were absent at the time of the studies, or because the interviewers did not ask about physical symptoms (or took them for granted), or is the emotional burden as—if not more—relevant to the overall asthma experience?

Asthma has sometimes been discounted as a serious illness, yet patients with even mild asthma can have severe flare‐ups that require hospitalization, and for some people, it can be life‐threatening.^97^ For the past 25 years, asthma has been clearly recognized as an inflammatory medical condition. Prior to this, asthma was often discredited as a “real” illness among health professionals and the community because it was believed to have a psychological basis.^98^ This was a particularly prevalent belief in the 1960s, during the childhood of some of the participants in the included studies. This may partly explain the feelings of judgement, either by others or oneself, that were reported in some of the studies in our synthesis.

Anticipating judgement from others was a significant feature influencing people's disease experience. The judgement could be real or perceived, and was of great concern to many participants across the studies. Our synthesis revealed that efforts to work around this involved a variety of cognitive and behavioural strategies, primarily concealment, selective disclosure, normalizing and taking proactive measures. Of note was the overall negative perception of asthma and efforts to hide it, which was of more or less priority depending on the person's context—their social, employment or home environment, alongside the frequency and intensity of their symptoms (requiring more or less effort to conceal). While asthma itself may range from extremely mild to extremely severe, understanding how individuals perceive their environment and respond accordingly is an integral step in addressing and explaining how and why individuals experience asthma differently. This is important because such perceptions could potentially be a barrier to seeking appropriate care, taking medications or seeking help.

Living with a chronic illness can be physically and emotionally challenging. Generally, our findings suggest that these challenges are harder to face when diagnosed as an adult. Those with childhood diagnosed asthma reported not really knowing a life without it and that managing their asthma was a routine experience. In contrast, negative emotions and feelings were typically felt by those who had been diagnosed as adults.^31^ Concerns about the future and controlling the condition may have been harder to accept.^31^ Thus, our findings suggest that age of diagnosis influences the amount of work involved in living with asthma.

People learn how to live with their asthma and develop personal preferences for management and care. Health‐care interactions can play a significant role in a person's illness experience, for example, receiving good quality or conflicting information, or encountering poor health professional knowledge. General practitioners (GPs) are central to the management of asthma in the community, and asthma represents one of the most frequently managed chronic problems by GPs.^99^ Our findings suggest that considerable work is invested by people with asthma to manage both the positive and negative interactions they encounter in the health‐care system. Unsatisfying health‐care experiences may significantly limit the work of individuals in trying to manage their asthma; some studies report associations between regular review with health professionals and medication adherence.^100^ Alternatively, some patients might develop low expectations for their asthma care. Patients in Cvetkovski et al.'s^63^ study reported being satisfied with their care despite health‐care providers perceiving the delivery of asthma care as suboptimal.

Clinical interactions can help people to form, maintain or re‐establish self‐identities and offer support for emotional load and social stigma. Unfortunately, for some individuals, their asthma experience was not validated by health‐care interactions. Some of our data suggested dismissive and negative judgmental comments from health‐care providers. These comments risk challenging a person's identity and undermining their self‐evaluation.

### 4.1 Clinical and policy implications

The topics of the included papers of this review were notably clinically focussed which is indicative of the type of studies that have been conducted in this area. Our higher‐order analysis about the work involved in living with asthma is about the psychological, emotional and social experience, particularly the burden of asthma for some. Our findings, with an emphasis on emotional experiences, suggest that what matters most to those with asthma is not necessarily what matters to clinicians. Similar findings have been made in other chronic illnesses.^101^ Clearly medical care is a central plank for managing chronic conditions, and it would be extremely detrimental to return to the perception of the 1960s that asthma was a psychological disease, but it is important for HCPs to consider the broader aspects of management, beyond that of symptoms and treatment, if our health‐care systems are to deliver patient‐centred care. Further in‐depth and focused investigation of these aspects is warranted, to gain first‐hand insight into the patient experience of asthma in order to direct evidence‐based efforts towards improving health‐care support systems.

Patients play an instrumental role in managing their own long‐term condition(s). Yet their capacity to manage their illness will inevitably depend on individual circumstances and the medical support they receive. Patient understanding of their illness and its treatment is a potentially modifiable mediator of adherence with medications and self‐management behaviours.^96^ Improved communication with health‐care professionals, including detailed understanding of the broader patient experience, is key.

There are implications emerging from this review for health‐care planners and policymakers to address, specifically improving supportive care within the health‐care system. More attention could be paid to the nuances and sensitivities surrounding self‐management which are relevant to the patient experience. HCPs need to be aware of how living with asthma must be accommodated within the context of individuals' life circumstances.

A review by Andrews identified that many self‐management programmes focus predominantly on medical management and overlook the social and psychological work of the asthma experience.^102^ Our review identified a notable gap in the literature, namely the role of social support in the lives of people who have asthma. Given the strong evidence base regarding the influence of social support on health management and outcomes,^103^ this is a key area deserving of further investigation. The findings of this review also highlight the importance of recognizing the complex and variable emotional experiences of those living with asthma—their perspectives and opinions, alongside medically driven considerations and measures.

### 4.2 Limitations

Some argue that it is difficult to synthesize studies that are carried out in disparate contexts and that attempting to do so ignores the rich detail that characterizes good qualitative research. However, like Britten et al.,^104^ we argue that it is important to realize the full contribution of qualitative research by synthesizing individual studies: “it is possible to make generalizations across qualitative research studies that do not supplant the detailed findings of individual studies—but adds to them.” This qualitative synthesis provides an in‐depth exploration and analysis of the spectrum of the patient's perspective of living with asthma.

These studies were mostly conducted in white populations. However, rates of asthma morbidity, mortality and acute resource utilization are highest among minority, non‐white, inner‐city populations, and of these groups, our knowledge and understanding of the experience of living with asthma remains poor.

People whose asthma is currently well controlled may be relatively under‐represented in existing literature, with 8 of 26 (31%) of the included studies recruiting emergency department attendees or patients admitted to hospital in order to investigate their specific experiences in these settings. However, emergency department visits are surprisingly common even in well‐controlled asthma; for example, in a large US survey, one in seven patients with well‐controlled asthma reported having an emergency department or urgent care visit in the previous year.^105^

## 5 CONCLUSION

We explored the existent qualitative empirical evidence reporting on adult experiences of living with asthma. To our knowledge, this is the first systematic review of the qualitative literature in this area. A key strength of this review was the methodology utilized to examine and interpret the findings from a diverse array of studies. We have brought to the forefront those aspects of the “work” of living with asthma that are important to those who have it, and highlight the unique nature of the personal experience of asthma and diversity in how individuals perceive, manage and relate to their experience. In so doing, we hope this work informs health‐care professionals in their care of patients with asthma and facilitates the delivery of a patient‐centred care approach to improving patient health and treatment outcomes.

## APPENDIX A

### A.1

ti=title, tw=text word

PsychInfo & Medline (variations made according to database requirements):

exp Asthma/
Asthma$.ti. or wheez$.ti,ab.
(asthmatic? or (asthma$ adj2 (chronic$ or patient?))).ab.
(lung disease or lung diseases).tw.
or/1‐4
exp adult/or exp aged/or middle aged/or young adult/
(patient* or inpatient*).tw.
or/6‐7
Qualitative Research/
ethnog*.tw.
phenomenolog*.tw.
participant observ*.tw.
constant compar*.tw.
focus group*.tw.
action research.tw.
qualitative stud*.tw.
(focus group* or interview*).tw.
(grounded adj (theor* or study or studies or research)).tw.
9 or 10 or 11 or 12 or 13 or 14 or 15 or 16 or 17 or 18
patient experience*.tw.
lived experience*.tw.
life experience*.tw.
patient perspective*.tw.
experience*.tw.
health experience*.tw.
living with asthma.tw.
personal experience*.tw.
illness experience*.tw.
quality of life*.tw.
20 or 21 or 22 or 23 or 24 or 25 or 26 or 27 or 28 or 29
5 and 8 and 19 and 30
limit 31 to (english language and humans)

Asthma OR wheez* (Cinahl, Embase, Medline)

Asthma* OR ASTHMA‐in DE OR wheez* (PsychINFO)(Embase)

exp *asthma/
(asthma$ or wheez$).ti.
(asthma$ adj3 (sever$ or chronic$ or primary or major)).ab.
or/1‐3 [Asthma]
(exp asthma/) and chronic disease? management.ti,ab.
4 and chronic disease? manag$.ti,ab.
or/5‐6 [Focussed Key Terms]

## APPENDIX B

### B.1

**Table B1:** Summary of included studies

Study/Country	Aims and Objectives	Method of analysis	Method and setting	Study population
Adams et al., 1997 UK^76^	Explore the perspective of patients with asthma on the use of preventer medications “To generate a hypothesis for the findings and extending concepts already used within the literature on chronic illness.”^76^	Thematic	In‐depth interviews (an interview guide was devised) carried out at the participant's own home	Sample (N): 30
				Age range: 19‐57
				Gender (% of females): 47
				Source: primary care
Al‐kalemji et al., 2014 Denmark^84^	Explore the perspectives of people living with asthma and how “coping mechanisms were influenced by health professionals and networks.”^84^	Thematic	Semi‐structured interviews conducted in either the participant's own home, work place or hospital	N: 10
				Age range: 36‐52
				Gender (% of females): 60
				Source: community population
Baptist et al., 2010 USA^62^	Reveal common challenges “older adults face to manage their asthma and provide age‐specific information to inform treatment and counselling/education decisions.”^62^	Thematic	Focus groups. Setting not stated	N: 46
				Age range: 65+
				Gender (% of females): 85
				Ethnicity: 23 identified as white, 20 as African American and 3 as other
				Source: primary and/or secondary care
Becker et al., 1993 USA^71^	None stated	Thematic	Three semi‐structured monthly interviews in research laboratory or at home	N: 95
				Gender (% of females): 62
				Source: not stated
Cvetkovski et al., 2009 Australia^63^	“Investigate the perceptions and attitudes of general practicioners, pharmacists and people with asthma, towards management of asthma.”^63^	Thematic	Semi‐structured interviews conducted in a small rural centre	N: 10
				Age: 18+
				Source: community pharmacies
Donald et al., 2005 Australia^64^	Why do adults living with “life threatening asthma report delaying treatment, and downplay the seriousness of their symptoms?”^64^	Thematic	Focus groups. Setting not stated but recruited from one of two metropolitan teaching hospitals.	N: 5
				Age range: 20‐42
				Gender (% of females): 60
				Source: patients admitted with life‐threatening asthma
Douglass et al., 2004 Australia^72^	“What do patients with asthma who seek emergency care look for in a doctor‐patient relationship?”^72^	Thematic	Semi‐structured interviews conducted in a city, rural and suburban hospital	N: 62
				Age range: 18‐69
				Gender (% of females): 69
				Source: ED attendees (same sample as Goeman 2002 and 2004)
Drummond 2000 UK^82^	How does asthma influence a patient's quality of life?	Thematic	Semi‐structured interviews in participants' homes	N: 22
				Source: primary care
Goeman et al., 2002 Australia^83^	“Explore the burden of asthma on the lives of people presenting to emergency department”^83^	Thematic	Semi‐structured interviews. Setting not stated	N: 62
				Age range: 18‐70
				Gender (% of females): 69
				Source: ED attendees (same sample as Douglass 2004 and Goeman 2004)
Goeman et al., 2004 Australia^73^	“Explore the reasons why patients recurrently present with asthma to emergency departments”^73^	Thematic	Semi‐structured interviews. Setting not stated	N: 62
				Age range: 18‐70
				Gender (% of females): 69
				Source: ED attendees (same sample as Douglass 2004 and Goeman 2002)
Goeman et al., 2007 Australia^65^	“Explore the perspectives of older people living with asthma, and the barriers which may exist and prevent optimal asthma care.”^65^	Thematic	In‐depth interviews. Setting not stated	N: 55
				Age range: 40% in their 60s
				Gender (% of females): 71
				Source: community population
Haughney et al., 2004 UK^77^	“Assess patient understanding of their asthma and their preferences regarding the delivery of asthma care and treatment.”^77^	Thematic	Semi‐structured interviews. Setting not stated.	N: 40
				Age range: 14‐65
				Source: community population
Health Experience Research Group (2015) UK^28^	Improve understanding of people with asthma experiences of health, illness and health care	Thematic	In‐depth, open‐ended questions followed by semi‐structured interviews conducted in participants' homes	N: 37
				Age range: 16‐73
				Gender (% of females): 65
				Source: community population
Hussein et al., 2002 UK^66^	“Explore 1) the knowledge, attitudes, perceptions, health beliefs and needs of those originally from India and Pakistan, and 2) their attitudes of self‐management plans.”^66^	Thematic	Two interviews were conducted: 1) semi‐structured and 2) focus groups. Setting not stated.	N: 60
				Age range: 16‐50
				Source: primary care
Janson et al., 1998 USA^78^	Explore how patients respond to acute asthma symptoms and understand why they would delay treatment	Thematic	Three monthly, semi‐structured interviews. Setting not stated	N: 95
				Gender (% of females): 62
				Source: primary and/or secondary care
Jones et al., 2008 UK^74^	Investigate the relationship between psychosocial factors, perception of life events and managing asthma in those who have been admitted and not admitted to hospital	Framework	Participants in hospital interviewed at the bedside and non‐hospital participants interviewed in their practice. Method not stated	N: 50
				Age range: 16+
				Gender (% of females): 64
				Ethnicity: 31 are white British, 22 Afro Caribbean, 8 Asians and 14 “others”
				Source: hospital and primary care (for comparison)
Lawson et al., 2014 USA^75^	Explore the reasons for “asthma‐related emergency department use among adults.”^75^	Thematic	Open‐ended and semi‐structured interviews conducted in a private area in a hospital	N: 26
				Age range: 18‐65
				Gender (% of females): 69
				Ethnicity: 21 African Americans
				Source: ED attendees
Loignon et al., 2009 Canada^67^	Understand how adults deal with their asthma, “perceive self‐management and develop self‐care strategies.”^67^	Interpretative phenomenological	In‐depth semi‐structured interviews conducted in participants' homes.	N: 24
				Age range: 27‐76
				Gender (% of females):58
				Additional information: Participants were all francophone, Quebec‐born individuals
				Source: ED and primary care (for comparison)
Mancuso et al., 2006 USA^86^	Investigate 1) the patients' views about exercise and lifestyle activities, and 2) whether these views varied depending on asthma characteristics.	Thematic	Open‐ended questions. Setting not stated	N: 60
				Gender (% of females): 88
				Ethnicity: 28 African Americans, 12 Latinos and 20 “white”
				Source: primary care
Munro et al., 1996 USA^80^	Characterize the experiences of participants with asthma in 1) the care they receive to manage their disease, 2) their confidence in asthma self‐management, 3) any barriers they identify in their asthma management and 4) any recommendations they would make in order to improve asthma care in their community	Thematic	Focus groups conducted in a conference room in a hospital	N: 8
				Age range: 19‐60
				Gender (% of females): 63
				Ethnicity: 7 African Americans and 1 Native American
				Source: ED attendees
Nunes et al., 2014 Portugal^81^	Explore 1) patients with asthma experience of engaging with health services and health‐care professionals and 2) how patients search for, interpret and act on medical information related to the condition	Narrative	Semi‐structured interviews conducted in hospitals.	N: 40
				Source: hospital inpatients
Oncel et al., 2012 Turkey^85^	Investigate the perceptions patients with asthma, have of the disease	Thematic	Participants were asked to write a letter. Setting not stated	N: 23
				Gender (% of females): 70
				Ethnicity: Turkish
				Source: primary and/or secondary care (all patients “in remission”)
Speck et al., 2014 USA^68^	Understand the barriers young African American adults, have with managing their asthma.	Thematic	Focus groups. Setting not stated.	N: 34
				Age range: 18‐30
	Finding strategies that may be used to improve self‐management and explore preferences for joining in asthma self‐management programme.			Gender (% of females): 68
				Ethnicity: young self‐identified African Americans
				Source: registry and community clinics
Steven et al., 2002 UK^79^	Identify factors which motivate patients with asthma self‐management and compare these to the British Thoracic Society (BTS) guidelines for asthma.	Thematic	In‐depth interviews at a place convenient to participants, such as their homes or the general practice surgery.	N: 23
				Age range: 20‐47
				Gender (% of females): 48
				Source: primary care
Taylor et al., 2014 Australia^69^	“Examine the influences of intergenerational relationships on beliefs, knowledge and practices about health and illness.”^69^	Thematic	Semi‐structured interviews conducted at a place convenient to participants.	N: 27
				Age range: 40+
				Gender (% of females): 70
				Source: community population
Van Mens‐Verhulst et al., 2004 Netherlands & Canada^70^	Explore the question: “How do mothers with asthma manage their illness?”	Thematic	Semi‐structured interviews conducted in participants' homes.	N: 8
				Age range: 31‐65
				Gender (% of females): 100
				Source: outpatient clinics (primary and/or secondary care)
